# A Large-scale Survey of CRF55_01B from Men-Who-Have-Sex-with-Men in China: implying the Evolutionary History and Public Health Impact

**DOI:** 10.1038/srep18147

**Published:** 2015-12-15

**Authors:** Xiaoxu Han, Yutaka Takebe, Weiqing Zhang, Minghui An, Bin Zhao, Qinghai Hu, Junjie Xu, Hao Wu, Jianjun Wu, Lin Lu, Xi Chen, Shu Liang, Zhe Wang, Hongjing Yan, Jihua Fu, Weiping Cai, Minghua Zhuang, Christina Liao, Hong Shang

**Affiliations:** 1 Key Laboratory of AIDS Immunology of National Health and Family Planning Commission, Department of Laboratory Medicine, The First Affiliated Hospital of China Medical University, Shenyang, China; 2 Collaborative Innovation Center for Diagnosis and Treatment of Infectious Diseases, Hangzhou, China; 3 AIDS Research Center, National Institute of Infectious Diseases, Tokyo, Japan; 4 Infectious Diseases Department, Beijing Youan Hospital, Capital Medical University, Beijing, China; 5 Sexually transmitted Disease and AIDS Department, Anhui Provincial Center for Disease Control and Prevention, Hefei, China; 6 Yunnan Provincial Center for Disease Control and Prevention, Kunming, China; 7 AIDS/STIs Prevention and Control Department, Hunan Provincial Center for Disease Control and Prevention, Changsha, China; 8 Sichuan provincial center for disease control and prevention, Chengdu, China; 9 Henan Provincial Center for Disease Control and Prevention, Zhengzhou, China; 10 Sexually Transmitted Disease and AIDS Prevention and Control Department, Jiangsu Provincial Center for Disease Control and Prevention, Nanjing, China; 11 Sexually transmitted Disease and AIDS Department, Shandong Provincial Center for Disease Control and Prevention, Jinan, China; 12 Infectious Disease Department, Guangzhou No. 8 Renmin Hospital, Guangzhou, China; 13 Sexually transmitted Disease and AIDS Department, Shanghai Municipal Center for Disease Control and Prevention, Shanghai, China

## Abstract

The HIV-1 epidemic among men-who-have-sex-with-men (MSM) continues to expand in China, involving the co-circulation of several different lineages of HIV-1 strains, including subtype B and CRF01_AE. This expansion has created conditions that facilitate the generation of new recombinant strains. A molecular epidemiologic survey among MSM in 11 provinces/cities around China was conducted from 2008 to 2013. Based on *pol* nucleotide sequences, a total of 19 strains (1.95%) belonged to the CRF55_01B were identified from 975 MSM in 7 provinces, with the prevalence range from 1.5% to 12.5%. Near full length genome (NFLG) sequences from six epidemiologically-unlinked MSM were amplified for analyzing evolutionary history, an identical genome structure composed of CRF01_AE and subtype B with four unique recombination breakpoints in the *pol* region were identified. Bayesian molecular clock analyses for both CRF01_AE and B segments indicated that the estimated time of the most recent common ancestors of CRF55_01B was around the year 2000. Our study found CRF55_01B has spread throughout the most provinces with high HIV-1 prevalence and highlights the importance of continual surveillance of dynamic changes in HIV-1 strains, the emergence of new recombinants, and the need for implementing effective prevention measures specifically targeting the MSM population in China.

HIV-1 infections among men-who-have-sex-with-men (MSM) continue to increase worldwide^1^. Though the prevalence of HIV-1 among the general adult population of most countries is low and/or declining, the number of MSM affected by HIV-1 infections continues to be disproportionately high^2^, and MSM in Asia are 19 times more likely than other MSM worldwide to become infected^3^. The prevalence of HIV-1 among MSM in China has reached 4.9% according to a recent cross-sectional survey of 61 cities in China^4^. Among newly diagnosed HIV infection cases, the proportion of MSM increased from 0.3% before 2005, to 29.4% in 2011.The regions with the highest HIV prevalence among MSM were Guizhou, Sichuan, Guangdong, Jiangsu, Henan, Liaoning and Beijing^4^,^5^.

Our recent nationwide survey revealed that multiple HIV-1 strains have been detected among MSM in China. Major strains include the 2 lineages of CRF01_AE and 1 lineage of CRF07_BC strains^6^. These three HIV-1 lineages account for more than 75% of HIV infections among MSM in nine major Chinese cities^6^. Co-circulation of multiple lineages of HIV-1 strains had led to the inevitable emergence of various forms of inter-genotype recombinants and of novel circulating recombinant forms (CRFs). In 2006, one CRF01_AE/B recombinant among MSM population was reported in Malaysia designated CRF33_01B. This was the first CRF identified among MSM in Asia. In contrast, among Chinese MSM, the first identified circulating recombinant form CRF55_01B was identified by our group in 2013^7^. Moreover, a recent paper reported that an outbreak prevalence of the CRF55_01B strains among MSM has formed in Shenzhen, southern China^8^.

In the present study, we discuss the first CRF (CRF55_01B) detected among MSM in China, specifically regarding its evolutionary history and public health impact, based on 975 newly diagnosed HIV-1 infected cases from a prospective HIV primary infection cohort and 2 cross-sectional surveys conducted on 11 provinces/cities between 2008 and 2013.

## Methods

### Ethics Statement

The study was approved by the ethics committee of the AIDS Research Center of China Medical University in Shenyang. All the methods involving human subjects were carried out in accordance with relevant approved guidelines and regulation. All study subjects provided informed consent regarding the provision of blood samples and HIV-genotype analyses.

### Study Subjects

Blood specimens were collected from a total of 975 newly diagnosed HIV-1 infected MSM in 11 provinces/cities across China between 2008 and 2013. These HIV infected cases included 3 sources: 2 cross-sectional studies were conducted in 2009–2010 and 2011–2012 respectively, including Liaoning, Beijing, Shandong, Henan, Anhui, Jiangsu, Shanghai, Sichuan, Hunan, Yunnan and Guangdong, representing different geographical locations and HIV prevalence across China. HIV-1 antibody positive cases were screened from 400 MSM in each province or city. Another source was a large-scale prospective HIV primary infection cohort in Shenyang, Beijing and Kunming (Shang and Wu, *et al*. unpublished data), recruitment for which was done by the categorical snowball-sampling method among high-risk MSM populations between 2008 and 2011. The case number in each site was listed in Table 1: Liaoning province (n = 263) in northeastern China, Beijing (n = 163) in northern China, Shandong province (n = 42), Anhui province (n = 136), Jiangsu province (n = 49) and Shanghai (n = 26) in eastern China; Henan province (n = 58) and Hunan province (n = 68) in central China; Sichuan (n = 63) and Yunnan (n = 67) provinces in southwestern China; and Guangdong province (n = 40) in southern China (Table 1).10 ml EDTA-3 K anti-coagulated peripheral blood samples was collected from each case, the plasma was separated within 6 hours after collection and frozen at −80 °C for further analysis.

### RNA extraction, partial *pol* gene amplification and sequencing

RNA was extracted from 280 μl of plasma using QIAamp® Viral RNA Mini Kit (Qiagen, Germany) in a final elution volume of 60 μl. The *pol* gene sequences (HXB2 2253–3318 nt) were amplified using a previously published method^9^. Briefly, partial gene sequences of the HIV-1 *pol* region (HXB2 2253–3318 nt) were reverse-transcribed, amplified with SuperScript^TM^ Polymerase One-Step RT-PCR System (Invitrogen), and subjected to nested amplification using GoTaq DNA Polymerase (Promega). PCR products were purified using QIAquick Gel Extraction Kit (Qiagen) and sequenced directly with ABI PRISM Bigdye Terminator Cycle Sequencing Ready Reaction Kit and the same primers used in the previous publication^10^.

### Single genome amplification and sequencing

The 5-kb 5′ and 3′ half-genomes were amplified from plasma RNA via single genome amplification and sequencing (SGAS) using SuperScript™ III Reverse Transcriptase and Platinum Taq DNA Polymerase High Fidelity (Invitrogen, State) as described in a previous publication^10^ to acquire a dominant single virus sequence from quasispecies. Amplicons were sequenced by Beijing Huada Scientific Corporation (Beijing) using internal walking primers.

### Phylogenetic tree and recombination breakpoint analyses

All sequences were screened by using the HIV BLAST tool to detect laboratory contamination. Valid sequences were aligned with HIV-1 reference strains from the Los Alamos HIV Sequence Database (http://www.hiv.lanl.gov). Alignment and manual editing were performed using Clustal X software (Version 2.0) and BioEdit software (Version 7.0, http://www.mbio.ncsu.edu/bioedit/bioedit.html), respectively. Phylogenetic analyses were performed using the neighbor-joining method based on the Kimura 2-parameter distance matrix and a transition-to-transversion ratio of 2.0 using the MEGA software version 5.0^6^. Tree topology was tested by bootstrap analysis with 1,000 replicates. HIV-1 recombinant analysis was carried out using Simplot (Version 3.5.1; http://sray.med.som.jhmi.edu/SCRoftware/simplot/) with a window size of 350 bp and a step size of 50 bp for near-full-length and half-genome sequences. A window size of 200 bp and a step size of 20 bp were used for *pol* gene fragments.

### Evolutionary analysis

Estimation of evolutionary rate and the time of the most recent common ancestor (tMRCA) for CRF01_AE lineages were performed as described previously^10^. Bayesian Markov chain Monte Carlo (MCMC) inference under the relaxed lognormal molecular clock was selected as a reliable mode for this analysis^11^. The MCMC chains were run 20 million times and sampled every 1000 steps. Bayesian MCMC output was analyzed using TRACER v1.5, and all parameters were estimated from an ESS > 200. The trees were summarized in a target tree using the Tree Annotator program and scanned using the Fig. Tree program1.3.1.

### GENBANK Accession Numbers

The near-full-length sequences reported in this article are available in GenBank under accession numbers JX574661 to JX574663, KF927150 to KF927151 and KC183777.

## Results

### Spread of CRF55_01B, the first CRF associated with transmission among MSM in 11 provinces in China

**Figure 1:**
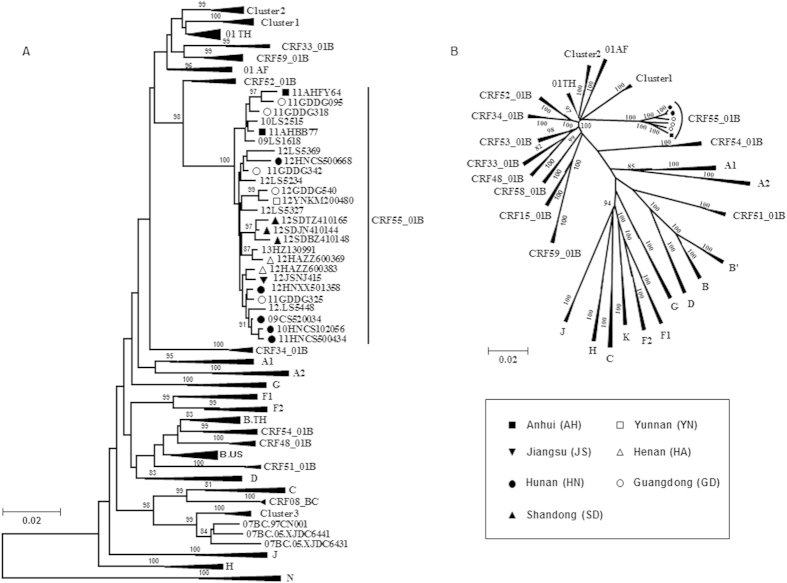
Neighbor-joining tree analysis of HIV-1 nucleotide sequences of the 1.1-kb pro-RT regions obtained from MSM in 11 cities in China. Neighbor-joining tree analysis of HIV-1 nucleotide sequences of the 1.1-kb pro-RT region (HXB2: 2253–3318nt) (n = 19) (**A**) and of NLFG sequences (HXB2: 790–9600 nt) (n = 6) (**B**) of CRF55_01B samples from MSM in various regions of China. The sequences were compared with representative CRF55_01B sequences published previously as well as all of the known subtypes/subsubtypes and CRFs reference sequences relevant to this study (http://www.hiv.lanl.gov/content/index). The sequences identified in different regions were shown with markers as follows: Anhui, the black rectangle; Jiangsu, black lower triangle; Hunan, black circle; Shandong, black upper triangle; Yunnan, the open rectangle; Henan, open lower triangle; Guangdong, black circle; The subtype reference sequences and previously published CRF55_01B sequences were shown with sequences ID.

**Figure 2:**
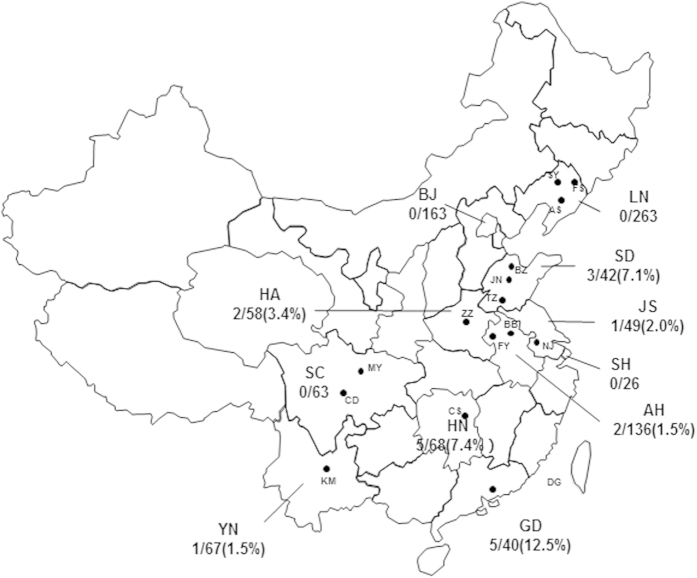
Map of the study sites and the distribution of CRF55_01B. This map of China shows the study sites (11 provinces) and the proportion of CRF55_01B among the HIV-1 strains identified among MSM at the respective study sites (province/city): Liaoning province/Shenyang, Anshanand Fushun (LN/SY AS and FS); Beijing (BJ); Shandong province/Jinan, Binzhou and Tengzhou (SD/JN,BZ,and TZ); Henan province/Zhengzhou (HA/ZZ); Jiangsu province/Nanjing (JS/NJ); Anhui province/Fuyang and Bengbu (AH/FY and BB); Shanghai (SH); Sichuan province/Chengdu and Mianyang (SC/CD and MY); Hunan province/Changsha (HN/CS); Guangdong province/Dongguan (GD/DG); Yunnan province/Kunming (YN/KM). This map is modified by the authors according to the free map template (http://wenku.baidu.com/) using MapInfo Professional 8.5(Pitney Bowes Inc.USA).

We determined the HIV-1 genotypes of a total of 975 samples collected from MSM in 11 provinces/cities in China using the nucleotide sequences of the ~1.1-kb *pol* (protease-RT) regions (HXB2: 2253–3318 nt) based on neighbor-joining tree analysis. The viral genotype distribution in among the MSM population was as follows: CRF01_AE (548, 56.2%), CRF07_BC (255, 26.2%), B/B’ (120, 12.3%), CRF55_01B (19, 1.9%); other CRFs and URFs (33, 3.4%) (Table 1). As shown in Fig. 1A, the CRF01_AE and CRF07_BC strains circulating among the MSM population formed three distinct monophyletic clusters^6^,^10^: CRF01_AE MSM clusters 1 (312, 32%) and 2 (229, 23.5%), and CRF07_BC cluster 3 (238, 24.4%) (Also as Table 1). Furthermore, we found an additional phylogenetic cluster (n = 19, 1.9%) with high statistical support for the cluster’s singularity (bootstrap value of 100%), belong to CRF55_01B reported by our group in 2013 (Fig. 1A). In this study, the CRF55_01B strains were detected in 7 out of 11 provinces/cities across China: the highest prevalence was found among MSM in Guangdong (12.5%, 5 of 40), Hunan (7.4%, 5 of 68) and Shandong provinces (7.1%, 3 of 42), followed by Henan (3.4%, 2 out of 58), Jiangsu (2%, 1 out of 49), Anhui (1.5%, 2 out of 136) and Yunnan (1.5%, 1 out of 67), but we noticed that no CRF55 strain has been detected in northern China (0 of 426) (Table 1, Fig. 2).

Recombination breakpoint analyses of the 1.1-kb *pol* (pro-RT) sequences of these strains showed that they contain a small subtype B segment within a CRF01_AE backbone (data not shown). To further characterize the recombinant structure of these strains, we determined NFLG sequences by using available plasma specimens from the 19 epidemiologically-unlinked MSM (Table 1). A total of 6 NFLG sequences from the different study subjects (three from Hunan, two from Guangdong, and one from Anhui) were successfully amplified and determined. As shown in Fig. 1B, neighbor-joining tree analysis of the NFLG sequences confirmed that these six strains indeed formed a distinct monophyletic cluster with a bootstrap value of 100%.

**Figure 3:**
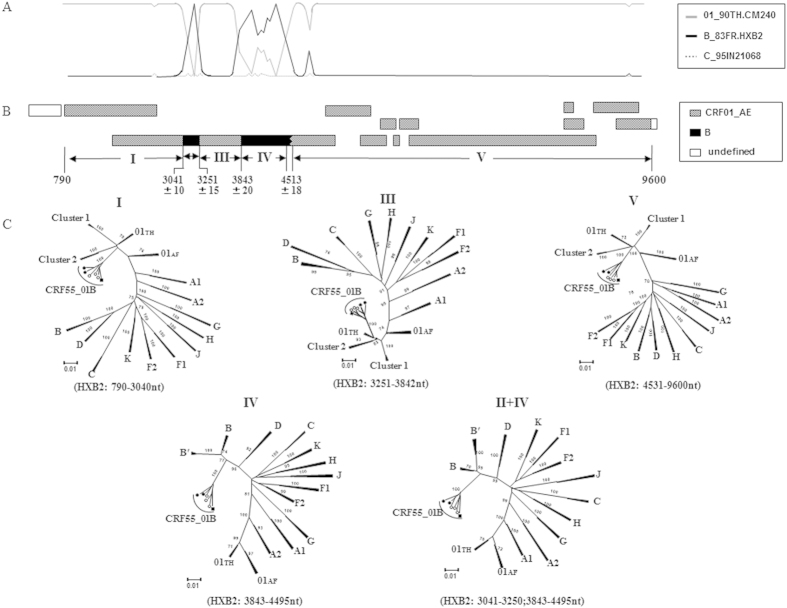
Recombination analyses of CRF55_01B. (**A**) Bootscanning plot analysis. Analyses were performed using CRF01_AE (90TH.CM240) and subtype B (83FR.HXB2) as parental subtypes, and subtype C (95IN21068) as the reference strain with a moving window of 350 nt and a step of 50 nt. (**B**) The deduced subtype structure. Black = subtype B (of US-European origin); gray = CRF01_AE; blank = sequence data not available. (**C**) Subgenomic phylogenies estimated using the neighbor-joining method from alignments representing regions I, III and V (CRF01_AE), and Regions II and IV (subtype B). Bootstrap scores greater than 70% are indicated at corresponding nodes. “01TH” = Thai CRF01_AE; “01AF” = African CRF01_AE; clusters 1 and 2 = CRF01_AE variants associated with transmission among MSM in China (An *et al*.; Kondo *et al*. JV).

Recombination breakpoint analyses revealed that these six strains had identical genome structure: two subtype B segments contained within a CRF01_AE backbone in the *pol* region (reverse transcriptase and integrase regions) (Fig. 3A,B). The recombinant structure is designated as to CRF55_01B^7^. To further confirm the subtype structure and to estimate likely parental lineages of CRF55_01B, we performed subregion tree analyses in which the HIV-1 genome was divided into five regions (denoted I, II, III, IV and V as illustrated in Fig. 3B). As shown in Fig. 3C, the CRF01_AE regions (Regions I, III and V) belonged to the Thai CRF01_AE radiation and did not belong to any other known CRF01_AE variants, including previously identified Chinese MSM clusters 1 and 2^10^. Similarly, subtype B regions (Regions II and IV) belonged to the typical subtype B of U.S.-European origin and were not related to the subtype B’ lineage (Thai variant of subtype B)^12^.

### Evolutionary characteristics of CRF55_01B

**Figure 4:**
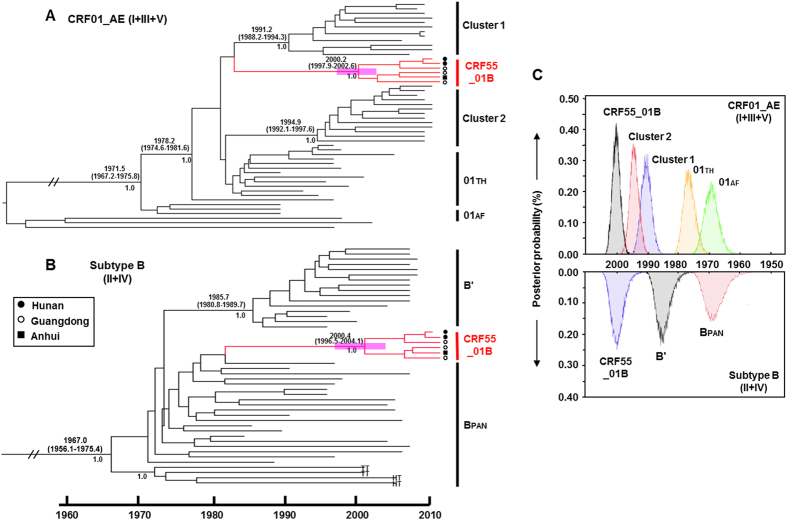
Maximum clade credibility (MCC) trees of CRF55_01B. The MCC tree was obtained by performing Bayesian MCMC analysis of the concatenated CRF01_AE (Regions I + III + V) (**A**) and the subtype B region (Regions II + IV) (**B**), using a relaxed clock model in GTR + G4 with a constant coalescent model. Analyses were implemented in BEAST v.1.6.0. HIV-1 subtype C sequences are used as an outgroup. The medians of tMRCAs with 95% highest probability density (HPD) (in parenthesis) and the posterior probability ( > 0.95) of the nodes relevant to this study were shown. (**C**) The distribution of the posterior probability of the estimated tMRCAs for CRF55_01B and related lineages: CRF01_AE lineages (top) and subtype B lineage (bottom).

To estimate the time of emergence of CRF55_01B, we performed Bayesian molecular clock analyses for the CRF01_AE regions [Regions I, III and V, and concatenated genome regions for CRF01_AE (Regions I + III + V)] and subtype B regions [Region II and the concatenated subtype B region (Regions II + IV)] (Fig. 4) using a relaxed molecular clock approach, respectively. Because the tMRCA estimations using individual or combined CRF01_AE regions [Regions I, III and V and the concatenated genome region for CRF01_AE (I + III + V)] yielded essentially similar results, we showed the maximum clade credibility (MCC) tree for only the concatenated CRF01_AE region (I + III + V) (Fig. 4A). For the subtype B region, because we were not able to obtain the tMRCA estimation with enough statistical support for Region II due to the shortness of the nucleotide sequence in this region (209nt), we provided the MCC tree for the concatenated subtype B segments (Regions II + IV) (Fig. 4B).

As shown in Fig. 4A,B, the estimated tMRCAs for the concatenated CRF01_AE regions (Regions I + III + V) and the concatenated subtype B regions (Regions II + IV) were 2000.2 [95% highest probability density (HPD): 1997.9, 2002.6] and 2000.4 95% HPD: (1996.5, 2004.1), respectively. The estimated tMRCAs for the CRF01_AE and subtype B regions were in agreement (see also Fig. 4C). This suggests that the recombination that generated CRF55_01B from parental lineages of subtype B and CRF01_AE occurred around the year 2000, consistent to the finding by Zhao *et al*. via the analysis of CRF55_01B pol fragments of Shenzhen MSM^8^. In contrast, the estimated tMRCAs for Chinese CRF01_AE MSM clusters 1 [1991.2 (1988.2, 1994.3)] and cluster 2 [1994.9 (1992.1, 1997.6)] are significantly older than those of CRF55_01B (Fig. 4A).

## Discussion

Our large-scale molecular epidemiologic survey (Table 1) revealed that CRF55_01B, originally identified among three epidemiologically-unlinked MSM in Guangdong and Hunan province in southern China^7^, disseminated widely among MSM in major cities southern, eastern and central China. Although the CRF55_01B strain only accounted for 1.9% (19 of 975) of HIV-1 infections among MSM in this study, the Guangdong province and Hunan province are still the regions with highest CRF55_01B prevalence (12.5 % and 7.4%, respectively) among the 11 provinces/cities, consistent with the regions where the CRF55_01B strains firstly reported. In addition to the above regions, we also found Shandong province, located in eastern China, with a relatively high CRF55_01B region prevalence (7.1%). Moreover, we detected CRF55_01B strains in MSM from Henan and Anhui in central China, Jiangsu in eastern China and Yunnan, southwestern China. The prevalence ranged from 1.5 to 3.4%. However, this new CRF has not been detected in northern China (0 of 426) (Table 1, Fig. 2). The above data implied CRF55_01B, the recently identified CRF, had spread widely. On the other hand, this was not a random sampling investigation and the sampling sizes were not proportional to the local HIV prevalence, no definitive conclusion could be got. However, in some regions, such as Liaoning, Beijing and Yunnan, a HIV primary infection cohort was included as well as 2 cross-sectional studies, the specimen outnumbered other regions, we can hardly detected CRF55_01B strains there (0–1.5%), implying CRF55_01B has little impact on the above regions. In summary, the apparent distribution differences suggested that the CRF55_01B might originate among MSM in southern Chinese provinces and then co-circulated in eastern and central Chinese provinces. A recent study on MSM in Shenzhen, southern China, further verified our estimation, Zhao *et. al*. reported CRF55_01B strains account for 9.2% of the 1072 *pol* sequences, and the earliest CRF55_01B samples was collected from MSM in Shenzhen as early as 2007^8^.

To date, a total of 11 CRFs comprising CRF01_AE and subtype B have been reported (http://www.hiv.lanl.gov/content/index): CRF15_01B and CRF34_01B from Thailand^13^,^14^; CRF33_01B, CRF48_01B, CRF53_01B, CRF54_01B and CRF58_01B from Malaysia^15^,^16^,^17^,^18^,^19^; CRF51_01B from Singapore^20^; CRF52_01B from Thailand and Malaysia^21^; and CRF55_01B and CRF59_01B from China^8^,^22^. Among them, CRF51_01B, CRF55_01B and CRF59_01B were first identified among MSM. These three CRFs comprise CRF01_AE and subtype B of U.S. European origin, while the other eight CRFs consist of CRF01_AE and the Thai variant of subtype B (referred as to subtype B’)^12^,^23^. This difference suggests that subtype B of U.S.-European origin entered first into the MSM populations of the aforementioned countries.

The emergence of CRF55_01B is a relatively recent event. As shown in Fig. 3, Bayesian molecular clock analyses revealed that the timing of the emergence of CRF55_01B is estimated to be around the year 2000 for both the CRF01_AE and subtype B regions. This timing indicates that CRF55_01B was indeed generated earlier this century via recombination between the CRF01_AE and subtype B strains co-circulating among MSM in southern China. This timing also makes CRF55_01B significantly younger compared to the other HIV-1 lineages associated with MSM transmission in China: CRF01_AE MSM cluster 1 (~1991) and cluster 2 (~1994) (Fig. 3)^6^. The founding effect due to the emergence of CRF55_01B well before HIV surveillance detected the rapid expansion of HIV infections among MSM in the mid-late 2000 s may explain the relatively high prevalence (~10% level) of this young CRF in some Chinese cities. Although CRF01_AE subregions of CRF55_01Bs still belong to Thai CRF01_AE, not CRF01_AE cluster1 or cluster 2 that are spreading in Chinese MSM^6^,^10^ (Fig. 3), and also these CRF55_01B are more fresh than the two CN-MSM CRF01_AE clusters, we believe that the more complex recombinants totally originated from Chinese MSM will emerge quickly, result from the frequent communication and co-circulating various HIV strains among MSM population.

The rapid upsurge of HIV infections among MSM in China is fuelled by high-risk behavior, including unprotected sex and exchanging sex for money, and inadequate knowledge about HIV among Chinese MSM^2^. This combination of ignorance and high-risk behavior makes MSM more vulnerable to super-infections. Therefore, the potential possibility of co-existing HIV-1 strains in individual MSM leads to the inevitable generation of new recombinant strains. Of the potentially many recombinant strains generated, only those that have spread widely via MSM transmission have come to be recognized as CRF(s). Indeed, several studies in China have begun to detect various recombinants and CRF candidates among MSM in different regions of China^24^,^25^,^26^,^27^,^28^,^29^. In our study, besides CRF55_01B, we found other potential CRF candidates among undefined recombinants (Table 1). We expect to identify additional new recombinant strains and CRFs among the Chinese MSM population.

In summary, we found that the novel recombinant CRF55_01B has disseminated widely among MSM in China. Our findings also detected the occurrence of diverse forms of potential recombinant strains affecting China’s MSM population, a result of the high-risk behavior exhibited by MSM that highlights the urgent need for implementing effective measures to reduce HIV-1 transmission in this population.

## Additional Information

**How to cite this article**: Han, X. *et al*. A Large-scale Survey of CRF55_01B from Men-Who-Have-Sex-with-Men in China: implying the Evolutionary History and Public Health Impact. *Sci. Rep*. **5**, 18147; doi: 10.1038/srep18147 (2015).

## Figures and Tables

**Table 1:** HIV-1 genotype distribution among MSM in China based on *pol* gene phylogenies.

Study site		Year of survey	N	CRF01_AE				CRF07_BC			B			CRF55_01B	Other CRFs and URFs
Region	Province			n	MSM cluster 1	MSM cluster 2	Other	n	MSM Cluster 3	Other	n	B	B′		
Northeastern	Liaoning (LN)	2008–2012	263	213	43	165	5	15	10	5	28	26	2	0	7
			(100)	(80.9)	(16.3)	(62.7)	(1.9)	(5.7)	(3.8)	(1.9)	(10.6)	(9.9)	(0.7)	(0.0)	(2.8)
Northern	Beijing (BJ)	2008–2011	163	81	57	24	0	30	28	2	51	50	1	0	1
			(100)	(49.7)	(35)	(14.7)	(0.0)	(18.4)	(17.2)	(1.2)	(31.3)	(30.7)	(0.6)	(0.0)	(0.6)
Eastern	Shandong (SD)	2009–2012	42	25	20	5	0	10	10	0	4	4	0	3	0
			(100)	(59.4)	(47.5)	(11.9)	(0.0)	(23.8)	(23.8)	(0.0)	(9.5)	(9.5)	(0.0)	(7.1)	(0.0)
	Anhui (AH)	2011	136	74	63	11	0	45	45	0	8	8	0	2	7
			(100)	(54.4)	(46.3)	(8.1)	(0.0)	(33.1)	(33.1)	(0.0)	(5.9)	(5.9)	(0.0)	(1.5)	(5.1)
	Jiangsu (JS)	2009–2012	49	29	21	7	1	10	9	1	3	3	0	1	6
			(100)	(59.2)	(42.9)	(14.3)	(2.0)	(20.4)	(18.4)	(2.0)	(6.1)	(6.1)	(0.0)	(2.0)	(12.3)
	Shanghai (SH)	2009–2012	26	17	13	4	0	4	4	0	5	5	0	0	0
			(100)	(65.4)	(50.0)	(15.4)	(0.0)	(15.4)	(15.4)	(0.0)	(19.2)	(19.2)	(0.0)	(0.0)	(0.0)
Central	Henan (HA)	2012	58	16	9	7	0	32	32	0	8	1	7	2	0
			(100)	(27.6)	(15.5)	(12.1)	(0.0)	(55.2)	(55.2)	(0.0)	(13.8)	(1.7)	(12.1)	(3.4)	(0.0)
	Hunan (HN)	2010–2012	68	28	24	4	0	33	30	3	1	1	0	5	1
			(100)	(41.2)	(35.3)	(5.9)	(0.0)	(48.5)	(44.1)	(4.4)	(1.5)	(1.5)	(0.0)	(7.4)	(1.5)
	Sichuan(SC)	2009	63	21	20	0	1	34	30	4	8	7	1	0	0
			(100)	(33.3)	(31.7)	(0.0)	(1.6)	(54.0)	(47.7)	(6.3)	(12.7)	(11.1)	(1.6)	(0.0)	(0.0)
Southwestern	Yunnan (YN)	2012	67	34	33	1	0	23	22	1	2	1	1	1	7
			(100)	(50.7)	(49.3)	(1.5)	(0.0)	(34.3)	(32.8)	(1.5)	(3.0)	(1.5)	(1.5)	(1.5)	(10.5)
Southern	Guangdong (GD)	2011–2012	40	10	9	1	0	19	18	1	2	2	0	5	4
			(100)	(25.0)	(22.5)	(2.5)	(0.0)	(47.5)	(45.0)	(2.5)	(5.0)	(5.0)	(0.0)	(12.5)	(10.0)
Total			975	548	312	229	7	255	238	17	120	108	12	19	33
			(100)	(56.2)	(32.0)	(23.5)	(0.7)	(26.2)	(24.4)	(1.7)	(12.3)	(11.1)	(1.2)	(1.9)	(3.4)

The genotypes were determined based on the phylogenetic analyses to partial pol gene (HXB2: 2253–3278 nt). The percentage of case number in each subtype or CRFs are shown in parentheses.
